# Work-Related Intervention Needs and Potential Occupational Outcomes among Medical Assistants: A Cross-Sectional Study

**DOI:** 10.3390/ijerph16132260

**Published:** 2019-06-26

**Authors:** Jessica Scharf, Patricia Vu-Eickmann, Jian Li, Andreas Müller, Peter Angerer, Adrian Loerbroks

**Affiliations:** 1 Institute of Occupational, Social and Environmental Medicine, Centre for Health and Society, Faculty of Medicine, University of Düsseldorf, Universitätsstr. 1, 40225 Düsseldorf, Germany; 2 Department of Environmental Health Sciences, Fielding School of Public Health; School of Nursing, University of California Los Angeles, Los Angeles, CA 90095, USA; 3 Institute of Psychology, Work and Organizational Psychology, University of Duisburg-Essen, Universitätsstr. 2, 45141 Essen, Germany

**Keywords:** health occupation, medical assistants, health care staff, working conditions, intention to leave, occupational outcomes

## Abstract

Medical assistants’ (MAs) working conditions have been characterized as precarious, and workplace-related intervention needs have been identified. However, strategies to change the MAs adverse working conditions are mostly seen on an individual level, including leaving the employer or even the profession. Since such intentions are antecedents of actual turnover, we aimed to quantify the potential link of reported unmet intervention needs with unfavorable occupational outcomes. Data were collected by means of a nationwide survey among medical assistants (n = 994) in Germany (September 2016–April 2017). The three subscales working conditions, reward from the supervisor, and task-related independence were derived from a 12-item instrument regarding work-related interventions needs (the independent variables). We used subscale-specific z-scores and a total needs z-score. The four outcome variables (i.e., intention to leave the employer, intention to leave the MA profession, choosing MA profession again, and recommending MA profession to young people) were dichotomized, and logistic regression analyses were performed and limited to MAs in employment (n = 887). We found that increasing needs according to the categorized total needs score were associated with increasing odds of all occupational outcomes. Needs pertaining to working conditions and reward from the supervisor were the strongest determinants of MAs’ consideration of leaving their employer or profession (Odds ratios: 1.55–2.61). In summary, our study identified unmet work-related intervention needs that are associated with unfavorable occupational outcomes. In light of staffing shortage in health care, the identified needs should be addressed to ensure that sufficient recruitment of junior staff in the profession of medical assistants remains feasible and that experienced staff is retained.

## 1. Introduction

The intention to leave (ITL) one’s employer has been identified as a strong predictor for actual staff turnover, which produces high costs due to the need to refilling vacant positions as well as the loss of organizational productivity and knowledge [1]. One sector for which the ITL has been identified as a particularly important issue is the health care sector. Due to expanding life expectancy and co-morbidity, patient numbers and thus also the pressure on health care staff is rising. Hospital nurses have especially been in the focus of recent research regarding predictors of the ITL [2,3,4,5,6,7,8,9,10,11]. A high level of job changes has however also been identified in staff working in outpatient care, such as the professional group of medical assistants (MAs; in German: Medizinische Fachangestellte) [12]. In Germany, MAs represent one of the largest occupational groups in the health care sector, along with physicians in in- or out-patient care and nurses working in hospitals. MAs usually provide basic clinical and administrative assistance in physician practices [13]. Under the supervision of physicians, MAs undertake multiple tasks that relate to patient care, such as taking blood samples, as well as administrative duties. 

The working conditions of MAs have been characterized as precarious in terms of, for instance, a low salary, irregular working hours, high job demands, and the need to handle unforeseeable incidents [12,14]. In this context, previous studies showed that MAs reported great dissatisfaction with their working conditions in relation to, among others, lacking influence at work and poor training possibilities [13,15,16,17]. Also, high work stress levels have been documented among MAs [17], for example, in terms of the well-known effort–reward imbalance (ERI) model [18]. While MAs are dissatisfied with many of their working conditions (e.g., salary, multitasking), it seems that they perceive very limited opportunities to change those conditions [17]. If they would want to talk about improving their working conditions, MAs would need to contact (and potentially criticize) their supervisor, since there are no employee representatives in small outpatient practices with few MAs [14]. In outpatient practices, the direct supervisors of MAs are commonly physicians who employ the MA and on whom they are thus financially dependent. As a consequence, most MAs dissatisfied with their working conditions report to primarily pursue individual-level strategies, which mainly pertain to exit strategies and thus the intention to leave the employer or the intention to leave the profession [14]. It has been shown among nurses—which in Germany is a health care profession comparable to MAs regarding patient contact and stressful working conditions—that job satisfaction is one of the most important determinants in predicting the intention to leave the employer or even the profession [1,9,17,19]. Work stress levels in previous studies among MAs and nurses have been mostly measured by the ERI model [2,14,20,21]. A previous study from our group documented that high work stress according to ERI was associated with increased odds of reporting the intention to leave the MA profession [14]. While the ERI and other dominant instruments that measure psychosocial working conditions are important tools in occupational health research, they do not cover to what extent employees want or expect certain working conditions to be actually modified. To fill this gap, we developed and used a tool which specifically measures MAs’ actual work-related intervention needs [22]. The resulting desired work-related intervention needs pertained to working conditions, reward from the supervisor and task-related independence [22]. Overall, high prevalences of unmet needs for work-related modifications have been identified among all participating MAs. 

The objective of the present study is to analyze the potential association of work-related intervention needs, as expressed by MAs, with ITL. To expand the scope of our study, we aimed to examine additional occupational outcomes beyond ITL, which likely also relates to MA staff shortage. Specifically, these outcomes may not only be relevant to staff retention (i.e., ITL), but also to the (in)ability to attract new staff (i.e., due to MAs who do not recommend to others to join their profession). Thus, in addition (i) to MAs’ intention to leave their profession and (ii) their intention to leave their current employer, we also considered (iii) whether MAs would choose their career path again and iv) whether they would encourage young people to enter the MA profession. 

## 2. Materials and Methods

### 2.1. Study Sample

We carried out a cross-sectional study and surveyed 994 MAs between September 2016 and April 2017. We included all MAs who consider themselves as MA and were either in MA training or were holding a professional degree as a MA. As the profession of medicals assistants is a state-approved and state-regulated vocational training, the occupational title is definite. The current analyses were restricted to MAs who reported to be in employment as MA (n = 887). To recruit participants, various communication channels were used, such as the members’ magazine of the Association of Medical Professionals (VMF e.V., which represents MAs), homepages of the Association of Statutory Health Insurance Physicians, and State Medical Association. Furthermore, physician offices or professional MA schools were contacted. The Ethics Committee of the Medical Faculty of the Heinrich-Heine-University of Düsseldorf approved this study. 

### 2.2. Independent Variables

Aside from sociodemographic and practice-related data, the questionnaire covered data on occupational activity and employment, job satisfaction, health status, involvement in patient care, and work-related intervention needs. Work-related intervention needs were measured by an instrument created by the study team. Briefly, potentially relevant items were derived from prior qualitative research [17], and the resulting item pool was then reduced and psychometrically evaluated [22]. This process yielded an instrument with 12 items which were grouped into three factors. The factor *working conditions* included five items pertaining to the needs of “working less hours”, “additional breaks”, “less multitasking”, “more staff”, and “different opening hours of the practice/clinic” (Cronbach’s alpha: 0.7). The factor *reward from the supervisor* comprised three items: “more appreciation for one’s work from the supervisor”, “more understanding of the supervisor”, and “a higher salary” (Cronbach’s alpha: 0.7). Four items made up the factor *task-related independence*: “more responsibility in my job”, “greater scope of action and freedom of choice”, “independently advising patients about their disease”, and “making home visits” (Cronbach’s alpha: 0.6). Initially, each item could be answered as follows: “Yes, I would like this”, “This need has already been met”, and “No, I do not need this”. This response format had been developed and proven useful in prior research [23]. To capture expressed wishes, the response categories were dichotomized into “Yes, I would like this” (scored as 1) versus “This need has already been met” or “No, I do not need this” (scored as 0). We calculated both factor-specific sum scores as well as a *total needs* score across all 12 items (Cronbach’s alpha for the 12 items: 0.7). 

### 2.3. Occupational outcomes

In terms of occupational outcomes, we collected the following data:

- Intention to leave one’s employer: “How often during the last 12 months have you thought about changing your employer?” with the choice among five answer categories: *never*, *a few times a year*, *a few times a month*, *a few times a week,* and *every day*. Both items measuring the intention to leave were used in previous studies to measure intentions to leave among nurses [21,24,25].
- Intention to leave one’s profession: “How often during the last 12 months have you thought about giving up your position as MA and starting another professional activity?” with the response categories *never, a few times a year, a few times a month, a few times a week,* and *every day*.
- In line with a previous study about the public perception of nursing professions [26], participants in our study were asked the following questions:
- Choosing MA profession again: “Would you, if you had to make the decision today, choose the MA profession again?”. This item covers the satisfaction with one’s job in retrospective assessment. The following answer categories were presented: *certainly*, *probably*, *rather not,* and *certainly not*.
- Recommending MA training: “I would recommend the training as MA to (younger) people” with the answer categories *I fully agree*, *I rather agree*, *I rather disagree,* and *I fully disagree*.

### 2.4. Statistical Analyses

Descriptive analyses were performed to show demographic information of the study population (see Table 1) and the prevalence of the outcome variables (see Table 2). In the next step, logistic regression models were run. The reported work-related intervention needs scores were set as independent variables. The total needs score was divided into tertiles (0–4 needs (the reference group), 5–7 needs, and 8–12 needs) for statistical analyses. Additionally, we ran subscale analyses with continuous variables of the total needs score as well as of all three factors. Doing so, we z-standardized the respective scores. 

The four outcome variables (i.e., considering to leave the employer, considering to quit the MA profession, had chosen the MA profession again as of today, and would recommend MA profession to young people) were dichotomized. In line with previous studies [21,24], the intention to leave the profession was considered present whenever participants reported that the corresponding thoughts arise at least several times a month. The same dichotomization was applied to the variable “intention to leave the employer”. The question addressing whether MAs would choose their profession again in retrospect was dichotomized as follows:

- yes: in any case, probably
- no: rather not, certainly not

The question measuring whether they would recommend the MA profession to young people was dichotomized as follows:

- yes: I fully agree, I rather agree
- no: I rather disagree, I fully disagree

Furthermore, the following sociodemographic and practice-related characteristics were included as confounders of the logistic regression models:

- Age (18–35 years, 36–45 years, ≥46 years)
- Sex (female, male)
- Gross salary (≤1499€, 1500€–1999€, ≥2000€)
- Employment status (full-time vs part-time/mini-job)
- Leadership position (yes vs no)
- Practice size (number of physicians and MAs)
- Practice location (major city >100,000 inhabitants, small city 20,000 to 100,000 inhabitants, rural area <20,000 inhabitants)

Associations between potential work-related intervention needs (i.e., independent variables) and occupational outcomes (i.e., dependent variables) were examined by logistic regression. The results of the logistic regressions are shown as odds ratio (OR) with 95% confidence intervals (CIs). Additionally, we performed Spearman’s partial correlation analyses as variables had not to be categorized for this analysis which may leads to a higher statistical power.

## 3. Results

Nearly all participants were female (98.4%) with an average age of 39.28 years (standard deviation (SD) = 11.43). Most of the MAs worked full-time (59.2%), had at least 11 years work experience (64.9%) and earned less than 2,000€ per month (58.5%). Almost half of the participants reported to hold a leadership position (48.0%). The practices in which MAs reported to work, were located in large cities (38.9%), small cities (42.0%) or in rural areas (19.1%), with an average practice size of 9.0 (SD = 5.4) employees (that is, MAs and physicians combined). On average, MAs reported to have worked 9.13 years (SD = 8.31) for their current employer and had been working in 3.10 (SD = 1.77) practices prior to their current employment.

The descriptive analysis of the outcome variables (see Table 2) shows that 30.7% had considered at least a few times a month to leave their employer, and 22.4% reported to have considered leaving the MA profession at least a few times during the last 12 months. Almost half of the MAs reported that if they were to choose again today, they would rather not or certainly not choose the MA profession again (49.4%). In total, 42.6% would (rather) not recommend to young people to pursue the MA profession for a career.

Table 3 provides estimates of the associations between work-related intervention needs and the intention to leave the employer or to leave the profession (separate models). We found that increasing needs according to the categorized total needs score were associated with increasing odds of reporting the consideration to leave one’s employer: Compared to those with four or less needs, those with five to seven needs had 4.9 times increased odds of reporting to consider leaving their employer (OR = 4.91; 95%-CI: 2.87–8.43), and the odds further increased when participants specified eight to twelve needs (OR = 9.28; 95%-CI: 5.35–16.12). Furthermore, the increase in one standard deviation of the total need score (2.62) was associated with a doubled odds of the intention to leave the employer (OR = 2.52). Overall, all intervention need subscales confirmed this patterns (all ORs significant and ≥1.5). Unmet needs pertaining to reward from the supervisor showed the strongest associations with the intention to leave the employer. We observed that the number of unmet needs was likewise associated with the intention to leave the MA profession (see also Table 3). Again, all subscales were associated with this outcome, in particular needs pertaining to reward from the supervisor (OR = 2.02, 95%-CI = 1.59–2.55).

In Table 4, the results for choosing the profession as MA again are presented. The odds of specifying that one would not choose the MA profession again was elevated in those with five to seven needs (OR = 3.68; 95%-CI: 2.40–5.64) and further increased in participants with eight to twelve needs (OR = 4.83; 95%-CI: 3.06–7.62) as compared to those specifying four or less needs. Overall, these patterns of associations were reproduced in subscale-specific analyses. Again, associations were particularly pronounced in case needs pertaining to reward from the supervisor or working conditions were reported.

As Table 4 further shows, both categories of the total needs score were also associated with reporting not to recommend the MA professional training (5–7 needs: OR = 3.65; 95%-CI: 2.38–5.62; 8–12 needs: OR = 5.44; 95%-CI: 3.44–8.59) compared to the reference category (0–4 needs). Strongest associations were found for reporting needs pertaining to working conditions as well as reward from the supervisor (see Table 4). The patterns of associations of the partial correlation analyses are consistent with our primary analyses (i.e., odds ratios) for all occupational outcomes. 

## 4. Discussion

In this study, we found that increasing occupational intervention needs of any type were associated with increasing odds of reporting the consideration to leave the field of profession or the employer, as well as not recommending the MA training to (younger) people and not choosing the training again in retrospect. Furthermore, a dose–response relationship between the amount of reporting any need and the occupational outcome was identified throughout all regression models. In analyses by types of needs (i.e., subscales), we observed that needs pertaining to reward from the supervisor were the most prominent determinant of MAs’ consideration to change their employer or profession. Needs pertaining to working conditions also showed significant associations, while the factor task-related independence was only associated with the occupational outcomes regarding the intention to leave the employer or the profession as well as choosing the MA profession again. 

### 4.1. Findings in Light of Prior Studies

With regard to the intention to leave the profession or the employer, it deserves mentioning that there is no prior study among MAs, except for a prior publication from our data set [14]. Thus, our findings can only be compared to studies among other health care professions. In the present study among MAs in Germany, 37.0% considered at least a few times per month to leave their employer, which is comparable to German midwives (≥40%) [27], but remarkably higher than among German nurses (≤15.0) [28,29]. While MAs seem to be even more unsatisfied with their working conditions compared to nurses, both seem a little more satisfied with their occupational tasks compared to the working conditions. Thus, the intention to leave the profession is lower among both professional groups compared to their reported intention to leave the employer (MAs: 22.4%; German nurses: 18.0% [28] and 10.8% [29]). While leaving the employer may produce costs due to the need to refill a position and training time, leaving the field of profession may have far greater consequences, that is, for the entire health care sector. Exits from the health care sector as a whole result in decreasing numbers of health care staff [1], as the Central Institute for Health Insurance in Germany (German: “Zentralinstitut für kassenärztliche Versorgung in Deutschland”) has found that already in 2015, every fifth practice was not able to refill vacant positions of MAs [30]. The limited availability of staff may lead to hiring of poorly or inadequately qualified staff or inability to refill positions, which may in turn, decreases the quality of care.

Our instrument measuring occupational intervention needs has been specifically developed for the MA profession based on prior qualitative research. This measurement of occupational intervention needs substantiates and expands the measurement of work stress, and helps to identify specific target areas for intervention. To understand how ITL in medical staff relates to psychosocial working conditions, several previous studies have used the ERI model [2,14,21] and other dominant models (i.e., job-demand-control model) [25]. The studies showed that those models are associated with both considering leaving the profession or the employer. Likewise, a previous study from our group analyzed and confirmed associations of ERI and the intention to leave the profession among MAs [14]. In our prior study, the ERI was measured by a 17-items questionnaire capturing to what extent certain working situations were applicable for the respondents. Those items refer to working situations, which might—only given that they are experienced—induce work stress. Some of the items are thus not measuring distress. For illustrative purposes, one may consider the ERI item *“I have a lot of responsibility in my job”.* This might be interpreted as a burden by some, but for others this might be a form of reward. Most importantly, agreement to that specific item does not necessarily imply the need for change of that working condition. In addition, some working conditions, which seem unequivocally stressful, may be considered an integral feature of one’s job as an MA, such as *working under a high stress level with multitasking*. In a previous study of our group, MAs highlighted that “one has to be made for the job” and that a high workload is a well-accepted job characteristic [17]; thus, while it is stressful, a reduction of the workload is less likely to be expected or expressed as a need. In addition, standardized work stress instruments may not capture the full scope of relevant factors from a specific working context. 

When we analyzed subcategories of occupational intervention needs, those needs pertaining to rewards from the supervisor showed the strongest associations with the intention to leave the employer. Previous studies have identified that the supervisors’ leadership qualities show great associations with the retention of staff [31,32,33]. In this context, MAs have already reported in earlier work not to receive the recognition from their supervisor that they expect [16] and that they experience low leadership quality [13]. Furthermore, recognition in terms of financial reward is often described as insufficient [12,15]. MAs are highly dependent on their supervisor for rewards, especially in primary care practices where their supervisor is also their employer. In Germany, such practices are usually considered small enterprises from a legal perspective, and this implies that staff representatives are lacking and employment protection is limited. This may be one reason why rewards from the supervisor were closely associated with the intention to leave the employer in our study: MAs tend to see the only way to change their situation is to leave their current employer [12]. Another determinant of the intention to leave the employer in our study was the factor working conditions. Correspondingly, also among physicians and nursing staff, working conditions were the main reasons for a planned or actual change of one’s employer or occupation, as reported in other studies [2,6,24,34]. While needs pertaining to reward from the supervisor and working conditions show the strongest associations with all the examined occupational outcomes, the factor task-related independence only showed few associations with the occupational outcomes. Earlier research showed that MAs are moderately satisfied with their freedom of choosing their working methods and also identified reward and workload as more relevant determinants [16,35].

Those two key factors (i.e., reward from the supervisor and working conditions) also showed pronounced associations with the intention to leave the profession. Those findings are in line with previous studies among nurses that have also identified low (financial) reward and high workload as the most important reasons for the intention to leave the profession [3,8,21,36]. 

While the intention to leave is related to the actual outflow of existing staff, we also sought to analyze the MAs’ perspective on their own job decision to choose the MAs profession for a career. Such views among currently employed MAs may contribute to attracting potential new staff: it has been documented that young people gather information that inform their career choices through various pathways [37], one of which are recommendations form their social networks. Similar findings have been made in a study among first-year nursing students, suggesting that knowing a working nurse or the recommendation of friends and family are the most important predictors for (not) choosing nursing training [38]. In light of these findings, it is a concern in terms of staff recruitment that 45.7% of the MAs in our study stated that they would not recommend the MA training to younger people which is in close keeping with a study among US nurses [26]. Although the comparability may be limited because registered nurses earn a higher salary compared to nurses, more suitable studies are missing. As Van Hoye and Lievens have observed, negative word of mouth decreases the effect of recruitment advertising on organizational attractiveness [39]. The perception MAs have of their profession is important for transporting a positive (or negative) public image of the profession, since they might (negatively) influence the career choice of their relatives, friend or even wider social network. To capture the satisfaction with the MA training and work field in retrospect, we additionally analyzed, if the participating MAs would choose the MA profession again as of today. In keeping with the study of Mills et al. among US nurses, the reported theoretical reelection of the MA profession is comparable to our study (54.0% vs 50.6%). While rejecting to choose the MA profession as of today for oneself may have several reasons, the negation of recommending the profession to others implies that the MAs assume that the work is demanding and that limited benefits of the profession will not be suitable for anyone. In our study, needs related to working conditions show the strongest associations with not recommending the profession as well as not choosing the MA profession again as of today. This finding is in line with previous research, which identified the current working conditions of MAs as precarious and demanding in terms of work stress [12,17]. This may be one reason for the already reported challenges regarding the recruitment and retention of MAs [30] as well as it may even worsen the current situation [26].

### 4.2. Implications

Our results can be seen as a needs assessment, which highlights promising target areas for interventions. Measures should also focus on structural processes, such as reducing or reorganizing workloads (e.g., defining responsibilities), since MAs do not see any preventive strategies or coping strategies that can be applied during working hours besides to simply continue with their occupational tasks [17]. Improved career prospects may increase MAs´ job satisfaction. As MAs wish to professionally develop and to take part in trainings [35], they could expand their scope of action (i.e., task-related independence) and through the assumption of additional tasks, improve their level of income. MAs working for general practitioners (almost half of our study population), for instance, may be offered the opportunity to take part in trainings as so-called care assistants in physicians’ practices (German: Versorgungsassistentin in der Hausarztpraxis: VERAH^®^), who additionally monitor and coordinate services, prevention, and case management, or perform home visits. The prerequisite for this is through an improved recognition and remuneration of MAs. Also, taking over such additional responsibility seems only useful if this does not imply a greater overall workload. However, as the extra costs for a higher remuneration of the MAs would have to be borne by the practice owners or rather by the profit of the practice, this may be challenging at current, since outpatient care in Germany and many other countries is subjected to budgeting and the additional expenditure on staff is not necessarily offset by a potential increase in profits. Nevertheless, since 2015, the employment of MAs as non-physician practice assistants and their services (e.g., home visits) can be billed outside the practices’ budget in Germany [40]. A prerequisite for the billing is a trained practice assistant (e.g., VERAH^®^) who is working at least 20 hours per week. Furthermore, during the past four quarters, 860 patients per physician (with full admission) or on average at least 160 patients who are older than 75 years per physician have to be treated in the outpatient practice [41]. Thus, in 2018, a structural surcharge of up to 2.536€ (maximum of 23,800 points, point value: 10.66 cent) per quarter can be generated which is supposed to cover the expenses on training, increased salary for the trained practice assistant and additional equipment such as mobile phone for home visits [42]. Nevertheless, for the MAs who are additionally trained as practice assistants and therefore can assume a broader scope of occupational tasks, physicians can generate billable services and higher salaries could be paid. 

As one need that has been reported by most of MAs pertains to higher income and MAs usually receive a gross salary far below the average income in Germany, political action is needed to retain staff and to ensure that sufficient staff is available on the labor market. In this context, salary increases in collective wage agreements to enhance the general attractiveness of the profession should be initiated. Still, it is not mandatory for medical practices and clinics comply with collective wage agreements [43]. Another aspect of rewarding the work of MAs relates to the quality of leadership of the physicians which seems to need addressing when one seeks to retain MA staff. Especially, the rewards that MAs received form their supervisor, in terms of recognition and remuneration, seem to have a direct impact on MAs’ job satisfaction [29], which, in turn, may affect the intention to leave the profession or the current employer. To ensure retention of MAs, efforts to improve leadership skills among physicians should be initiated. On a national level, the Federal Medical Association (“Bundesärztekammer”) in Germany has developed a curriculum on medical leadership which is designed for medical professionals from inpatient as well as outpatient care in leadership positions, but which is not mandatory for future physicians or practitioners, yet. Previous studies found that leadership affects employees’ health at the workplace through influencing the working climate, promoting healthy work designs, or functioning as a role-model regarding healthy behavior [44]. In contrast, poor leadership appears to be associated with adverse health outcomes (e.g., burnout) [31,32,33,45]. Therefore, it might be recommendable to implement development programs for physicians. To improve the understanding and communication across professional groups, the inclusion of MAs within these programs is also recommended [46]. 

As a previous study of our group has shown, the prevalence of work-related intervention needs is high among MAs [22]. Furthermore, there was no evidence of associations between the unmet work-related intervention needs and various individual or practice related factors. This suggests that there are no specific subgroups that need to be taken into account when planning interventions. Instead, all MAs should be targeted [22]. 

### 4.3. Strengths and Limitations

Our study is cross-sectional and therefore no conclusions can be drawn related to potential causality of associations; longitudinal studies are thus needed to disentangle potential temporal sequences. The recruitment process involved various pathways, therefore, we are unable to report the number of contacted MAs and thus no response rate can be estimated. Additionally, possible selection effects in terms of the overrepresentation of either dissatisfied MAs or committed MAs cannot be excluded. Concerning representativeness, it is reassuring though that characteristics of our sample are comparable to the characteristics of another study among MAs in Germany [47]. Additionally, the recruited study population is almost identical to corresponding characteristics according to data from Federal Statistical Office on the MA population in Germany (i.e. age, gender, and working arrangements) [48].

The outcome variables regarding the intention to leave either the profession or the employer have already been used in previous studies [21,25] and have been identified as strong predictors of actual turnover [49]. The remaining two outcome variables relating to choosing the MA profession again and recommending the MA training to others have been adjusted for MAs following a study of Mills et al. [26] which analyzed the career satisfaction of nurses.

The instrument measuring work-related intervention needs was devised based on prior qualitative research by our group [17] and psychometric analyses [22]. It may therefore be particularly suitable for MAs in Germany, and its validity could be limited in other countries and settings.

## 5. Conclusions

Our study identified unmet work-related intervention needs that are associated with occupational outcomes, such as the intention to leave the profession or the employer. In light of the personnel shortages, the identified needs should be addressed (e.g., by political action) to ensure that sufficient recruitment of junior staff in the profession of medical assistants remains feasible and that experienced staff is retained. 

## Figures and Tables

**Table 1:** Description of the sample (n = 887).

Characteristics	
Age, mean (standard deviation)	39.3 (11.4)
18–35, n (%)	264 (30.1)
36–45, n (%)	312 (35.5)
≥46, n (%)	302 (34.0)
Female, n (%)	865 (98.4)
Marital status, n (%)	
In a partnership	445 (50.7)
Single	433 (49.3)
Highest school degree, n (%)	
Low ^1^	52 (5.9)
Intermediate ^2^	653 (73.6)
High ^3^	170 (19.4)
Gross salary (€), n (%)	
≤1499	291 (33.6)
1500–1999	216 (24.9)
≥ 2000	360 (41.5)
Years in job, mean (SD)	17.3 (11.3)
Years in job, n (%)	
0–10	293 (35.1)
11–20	236 (28.3)
≥21	305 (36.6)
Practice type, n (%)	
General practitioner	345 (47.7)
Specialist	379 (52.5)
Employment status, n (%)	
Full-time	510 (59.2)
Part-time/Mini-job	351 (40.8)
Leadership position (yes), n (%)	421 (48.0)
Number of MAs in the employing practice, n (%)	
1–3	288 (34.6)
4–6	317 (38.1)
≥7	227 (27.3)
Number of practitioners in the employing practice, n (%)	
1	262 (31.5)
2	250 (30.0)
≥3	320 (38.5)
Practice size, mean (SD)	9.0 (5.4)
Practice location, n (%)	
Large city	343 (38.9)
Small city	370 (42.0)
Rural area	168 (19.1)

^1^ Low: secondary modern school qualification (‘Haupt-/Volksschulabschluss’); ^2^ Intermediate: secondary school level I certificate (‘Mittlere Reife’); ^3^ High: general qualification for university entrance (‘Abitur’) or entrance qualification limited to universities of applied sciences (‘Fachhochschulreife’).

**Table 2:** Descriptive analysis of occupational outcome variables.

Occupational Outcomes		n (%)
Thinking about leaving the employer	Never	278 (31.4)
	Few times a year	335 (37.9)
	Few times a month	136 (15.4)
	Few times a week	94 (10.6)
	Every day	42 (4.7)
Thinking about leaving of the MA profession	Never	363 (41.1)
	Few times a year	323 (36.6)
	Few times a month	96 (10.9)
	Few times a week	67 (7.6)
	Every day	34 (3.9)
Would choose the MA profession again today	Certainly	152 (18.2)
	Probably	271 (32.4)
	Rather not	258 (30.8)
	Certainly not	156 (18.6)
Would recommend MA profession to young people	I fully agree	114 (13.0)
	I rather agree	364 (41.4)
	I rather disagree	281 (32.0)
	I fully disagree	120 (13.7)

**Table 3:** Results of intention to leave the employer and profession *.

Independent Variables			Intention to Leave the Employer		Intention to Leave the Field of Profession	
	Needs (n)	Standard deviation	Odds Ratio	95%- Confidence interval	Odds Ratio	95%- Confidence interval
Total needs score	0–4		Ref.		Ref.	
	5–7		4.91	2.87–8.43	3.80	2.06–7.00
	8–12		9.28	5.35–16.12	6.16	3.32–11.43
		2.62	2.52	2.03–3.13	2.15	1.71–2.71
Needs working conditions		1.58	1.67	1.39–2.02	1.55	1.26–1.91
Needs reward from the supervisor		1.05	2.61	2.08–3.27	2.02	1.59–2.55
Needs task-related independence		1.17	1.54	1.30–1.83	1.46	1.22–1.75

* adjusted for age, sex, gross salary, employment status, leadership position, practice size.

**Table 4:** Results of choosing MA profession again and recommending the occupational training *.

Independent Variables			Not Choosing MA Profession Again		Not Recommending Occupational Training as MA	
	Needs (n)	Standard deviation	Odds Ratio	95%- Confidence interval	Odds Ratio	95%- Confidence interval
Total needs score	0–4		Ref.		Ref.	
	5–7		3.68	2.40–5.64	3.65	2.38–5.62
	8–12		4.83	3.06–7.62	5.44	3.44–8.59
		2.62	2.09	1.73–2.52	2.04	1.70–2.45
Needs working conditions		1.58	1.74	1.46–2.07	1.88	1.58–2.25
Needs reward from the supervisor		1.05	1.73	1.46–2.05	1.80	1.52–2.14
Needs task-related independence		1.17	1.36	1.15–1.61	1.14	0.97–1.33

* adjusted for age, sex, gross salary, employment status, leadership position, practice size.
